# Orthobunyavirus Ultrastructure and the Curious Tripodal Glycoprotein Spike

**DOI:** 10.1371/journal.ppat.1003374

**Published:** 2013-05-16

**Authors:** Thomas A. Bowden, David Bitto, Angela McLees, Christelle Yeromonahos, Richard M. Elliott, Juha T. Huiskonen

**Affiliations:** 1 Oxford Particle Imaging Centre, Division of Structural Biology, Wellcome Trust Centre for Human Genetics, University of Oxford, Oxford, United Kingdom; 2 Biomedical Sciences Research Complex, School of Biology, University of St Andrews, St Andrews, Fife, Scotland, United Kingdom; NIH, United States of America

## Abstract

The genus *Orthobunyavirus* within the family *Bunyaviridae* constitutes an expanding group of emerging viruses, which threaten human and animal health. Despite the medical importance, little is known about orthobunyavirus structure, a prerequisite for understanding virus assembly and entry. Here, using electron cryo-tomography, we report the ultrastructure of Bunyamwera virus, the prototypic member of this genus. Whilst Bunyamwera virions are pleomorphic in shape, they display a locally ordered lattice of glycoprotein spikes. Each spike protrudes 18 nm from the viral membrane and becomes disordered upon introduction to an acidic environment. Using sub-tomogram averaging, we derived a three-dimensional model of the trimeric pre-fusion glycoprotein spike to 3-nm resolution. The glycoprotein spike consists mainly of the putative class-II fusion glycoprotein and exhibits a unique tripod-like arrangement. Protein–protein contacts between neighbouring spikes occur at membrane-proximal regions and intra-spike contacts at membrane-distal regions. This trimeric assembly deviates from previously observed fusion glycoprotein arrangements, suggesting a greater than anticipated repertoire of viral fusion glycoprotein oligomerization. Our study provides evidence of a pH-dependent conformational change that occurs during orthobunyaviral entry into host cells and a blueprint for the structure of this group of emerging pathogens.

## Author Summary

Orthobunyaviruses comprise a group of emerging arboviruses within the *Bunyaviridae*, the largest family of membrane-containing viruses. In spite of the continued medical impact upon human and animal health, little is known about orthobunyavirus structure or the process of host cell entry. Here, we address this paucity of information through electron cryo-microscopy analysis of Bunyamwera virus, the prototypic representative of this genus. We reveal that Bunyamwera virions are pleomorphic and display locally-ordered lattices of viral glycoprotein spikes on the envelope surface. The three-dimensional structure of the glycoprotein spike was resolved to 3.0-nm resolution. The spike is composed of the attachment and fusion glycoproteins and comprises a unique tripodal organization. This glycoprotein arrangement contrasts those observed in other virus families. Consistent with the established pH-dependent mechanism of membrane fusion during host cell entry, we provide evidence for the disruption of this tripodal assembly upon exposure to acidic environments. These data constitute a blueprint for orthobunyavirus architecture and support a case for broadened structural diversity within the *Bunyaviridae* family.

## Introduction

The family *Bunyaviridae* constitutes the largest known group of viruses containing RNA genomes [1]. Over 350 bunyaviruses have been identified and are divided into five genera: *Hantavirus*, *Orthobunyavirus*, *Nairovirus*, *Phlebovirus*, and *Tospovirus* [2], [3]. The members of this genetically diverse group are distributed world-wide and infect vertebrates, invertebrates, and plants. With the exception of hantaviruses, these viruses are arthropod-borne.

Approximately fifty bunyaviral species have been classified within the *Orthobunyavirus* genus [3]. Many orthobunyaviruses are zoonotic and have emerged as major human pathogens. Oropouche virus (OROV), for example, is the causative agent of a febrile disease known as Oropouche fever and has caused over thirty major epidemics throughout Central and South America, infecting more than half a million people over the past forty years [4]–[6]. La Crosse virus (LACV), which causes severe encephalitis and aseptic meningitis mostly in children, is endemic throughout Southern and Midwestern regions of the United States and recently outbreaks have been reported outside normal geographic areas of concern [7]–[10]. Orthobunyavirus infections have also been of significant impact to animal husbandry. Schmallenberg virus (SBV), first detected in late 2011, is a notable example, and is now prevalent throughout Europe, causing severe and often fatal infections in newborn sheep, cattle, and goats [11]–[13]. There are no existing therapeutics to treat or prevent orthobunyavirus infection.

Bunyamwera virus (BUNV), a mosquito-borne pathogen first isolated in Uganda in 1943 [14], is designated the prototypic representative of the *Orthobunyavirus* genus [1]. BUNV is an ideal model by which to study orthobunyavirus pathobiology as it constitutes a limited threat to human and animal health and is readily amenable to reverse genetics systems [15]. Similar to other orthobunyaviruses, BUNV particles comprise a lipid-bilayer envelope that encapsulates a negative-sense, single-stranded RNA genome. The genome is divided into three segments: L, M, and S. The L segment encodes the RNA polymerase, the S segment the nucleoprotein, and the M segment the glycoprotein precursor. The glycoprotein precursor is cleaved by host proteases into a non-structural protein (NSm) and two type I integral transmembrane glycoproteins, Gn and Gc, which associate to function as the attachment and fusion machinery required for host cell entry [16], [17]. It remains unclear which of the BUNV glycoproteins mediates attachment. Similarly, the functional cellular receptor(s) utilized during viral entry is unknown [18]. However, Gc is predicted to form a pH-dependent class-II fusion glycoprotein structure [19], [20]. Furthermore, the Gn and Gc glycoproteins are thought to heterodimerize in the endoplasmic reticulum and further assemble to form higher order assemblies or ‘spikes’ on the virion surface [21].

The Gn glycoprotein of BUNV is approximately 34 kDa in size and consists of an N-terminal ectodomain (205 residues), a transmembrane region (41 residues), and a long C-terminal cytoplasmic tail (55 residues). The Gc glycoprotein (109 kDa) is considerably larger and includes an N-terminal ectodomain (910 residues), a transmembrane region (23 residues) and a shorter C-terminal cytoplasmic tail (23 residues). The sequences of the BUNV glycoproteins are conserved with those of more pathogenic relatives. For example, BUNV shares 54% and 53% sequence similarity with the M segment of SBV and OROV, respectively, indicating a high degree of structural similarity between viruses [22].

Despite the continued impact on global health and economy, little is known about orthobunyavirus structure. Here, we address this paucity of information through electron cryo-microscopy of the prototypic BUNV. We show that BUNV forms roughly spherical, pleomorphic virions. The glycoprotein spikes, protruding from the virion membrane, form locally ordered lattices, and become disordered upon exposure to an acidic environment. Using electron cryo-tomography and sub-tomogram averaging, we generated a three-dimensional model of the glycoprotein spike in the prefusion state to 3.0-nm resolution. This structure was further verified by reconstruction using 2-D single particle averaging. In contrast to the other genera of the bunyavirus family, we observe that the BUNV spike forms a distinctive tripod-like structure. Our data provide a structural blueprint for this unique group of emerging viruses.

## Results and Discussion

### Preparation of Bunyamwera Virus

For virus propagation and purification, baby hamster kidney (BHK) cells were infected with BUNV. Medium containing mature [23], secreted virus particles was purified by ultracentrifugation through a sucrose cushion, resuspended at neutral pH (pH 7.4), and vitrified by rapid freezing. Sample purity was confirmed by SDS-PAGE analysis; the structural proteins (N, Gn, and Gc) were clearly visible and corresponded to their calculated molecular masses (Figure S1). The L protein was barely visible in the gel, probably due to low copy number in the virion [2].

### The Pleomorphic Ultrastructure of Bunyamwera Virus

**Figure 1:**
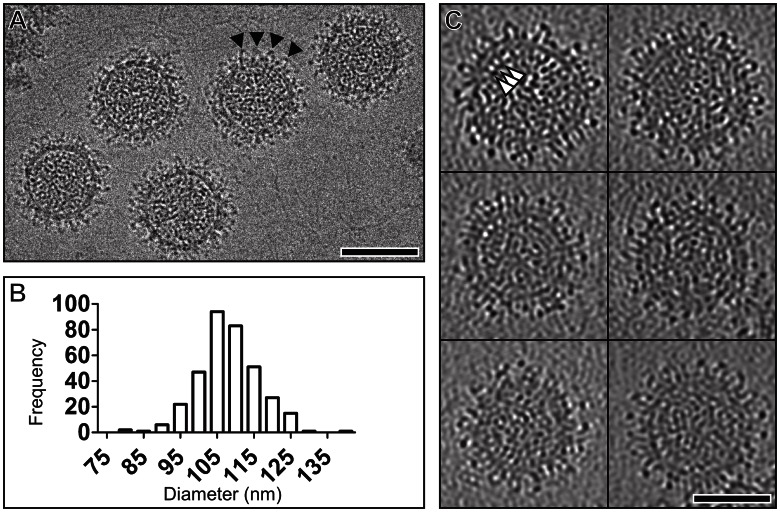
Electron cryomicroscopy of Bunyamwera virus (BUNV). (A) Electron cryomicroscopy image of BUNV virions taken at 5 µm under-focus. Triangles point to glycoprotein spikes. Scale bar, 100 nm. (B) Histogram displaying the variation in diameter (nm) of the BUNV particles. (C) 5-nm thick computational sections from high defocus (−7 µm) tomographic reconstructions of six BUNV particles. Triangles point to bridging density between the membrane and RNP density. Scale bar, 50 nm.

The shape and size of BUNV virions were analyzed by electron cryo-microscopy of 350 particles (Figure 1A). Our analysis revealed that BUNV forms roughly spherical virions. To assess if the size of BUNV particles was constant, as would be expected for an icosohedrally symmetric virus, we measured the particle size variation. The average diameter of the virions was 108±8 nm (Figure 1B). For comparison, we measured the size variation of an icosahedrally symmetric bunyavirus with a similar size, Rift Valley fever virus (RVFV) (Figure S2) [24]–[26]. We observed that the variation in diameter was smaller for RVFV (102±3 nm) than for BUNV. On the other hand, the diameter of LACV, another orthobunyavirus, has been reported to range between 75 and 115 nm [27]. These data suggest that orthobunyaviruses are unlikely to be icohedrally symmetric.

The structure of BUNV was further examined by electron cryo-tomography. We collected single-axis tilt series and reconstructed the three-dimensional volume of twenty-nine BUNV particles (Figure 1C). In agreement with the 2-D images, the tomographic reconstructions revealed BUNV particles exhibiting similar variation in diameter (±9 nm), confirming that unlike RVFV, BUNV is unlikely to be icosohedrally symmetric but instead pleomorphic. Structural pleomorphism is a common feature amongst genera of the *Bunyaviridae* family. In addition to being present in orthobunyaviruses, it has also been reported for nairoviruses [28] and hantaviruses [29], [30]. However, unlike Tula hantavirus, which forms both spherical and elongated virions [29], we did not observe any elongated BUNV virions. Pleomorphism renders single particle averaging methods used for structure determination of entire virions [31] incongruous for orthobunyaviruses.

Our micrographs and tomographic reconstructions revealed distinctive spikes, putatively corresponding to Gn–Gc glycoprotein heterodimers [16], protruding from and covering the entire virion membrane (Figure 1A,C). In contrast to previously characterized hantaviruses, we do not observe any significant areas of the membrane devoid of spikes [29]. The membrane surrounds an inner ribonucleoprotein (RNP) core. Density connecting the RNP core to the membrane likely corresponds to the sites of interaction between the intraviral glycoprotein cytoplasmic tails and the nucleoprotein, as proposed for another bunyavirus, Uukuniemi virus (UUKV) [26]. As bunyaviruses lack a matrix protein, this interaction has been suggested to be important for genome packaging [32]. Similarly to that observed in UUKV, the nucleoprotein and tri-segmented genome appears as a punctuated moiety, fully occupying the inner core of the virion (Figure 1C) [26].

### Tripodal Architecture of the BUNV Glycoprotein Spike

**Figure 2:**
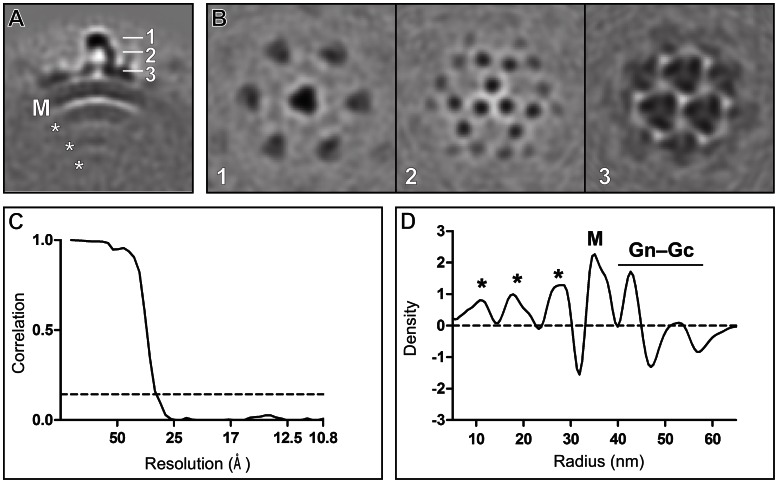
Subtomogram averaging of BUNV. (A) A 5-nm thick section through averaged BUNV density. Three layers of RNP (*), membrane (M), and glycoprotein spike complex (1–3) are labelled. (B) 5-nm thick cross-sections taken at the radii indicated in panel *A*. (C) Resolution assessment by Fourier shell correlation. The dashed line corresponds to a correlation threshold of 0.143 and intersects the plot at 3.0 nm. (D) A radial density plot of the averaged structure. The different layers are labelled as in panel *A*. The Gn–Gc glycoprotein layer is labelled with a bar. On average, the RNP density is the greatest at the distance of 11, 19, and 27 nm from the virion centre.

The glycoproteins Gn and Gc facilitate attachment and fusion with the host cell and form heterodimeric spikes on the virion surface [16]. We sought to resolve the structure of the BUNV Gn–Gc glycoprotein spikes, the associated membrane bilayer, and RNP components using sub-volume averaging of the tomographic reconstructions. Approximately 2,000 sub-volumes were aligned to generate an averaged structure (Figure 2A, B). The structure reveals a spike extending from the membrane (Figure 2A). The two leaflets of the 6-nm thick bilayer are clearly discernible (Figure 2A). The resolution of the structure was 3.0 nm, as determined by Fourier shell correlation (FSC) calculated between two independent sets of data (Figure 2C). To validate this structure, we further reconstructed the BUNV glycoprotein spike using the single-particle approach established by Battisti *et al.* to study the glycoprotein structure of Hantaan virus [30]. The application of this independent method resulted in a trimeric glycoprotein spike structure nearly identical to that derived by our subtomogram averaging (Figure S4; see Materials and Methods).

A radial density distribution calculated from our structure derived by subtomogram averaging revealed clear density for the glycoprotein spikes (radii between 37–54 nm), membrane bilayer (32–37 nm), and RNP (located preferentially at radii 11, 19, 27 nm) (Figure 2D). Although RNP is likely to be distributed throughout the virion, the three preferential radial peaks of RNP may be due to packing of the N protein and single-stranded RNA as well as the putative interaction between the N protein and the cytoplasmic tails of the glycoprotein spike, as suggested for UUKV [26].

**Figure 3:**
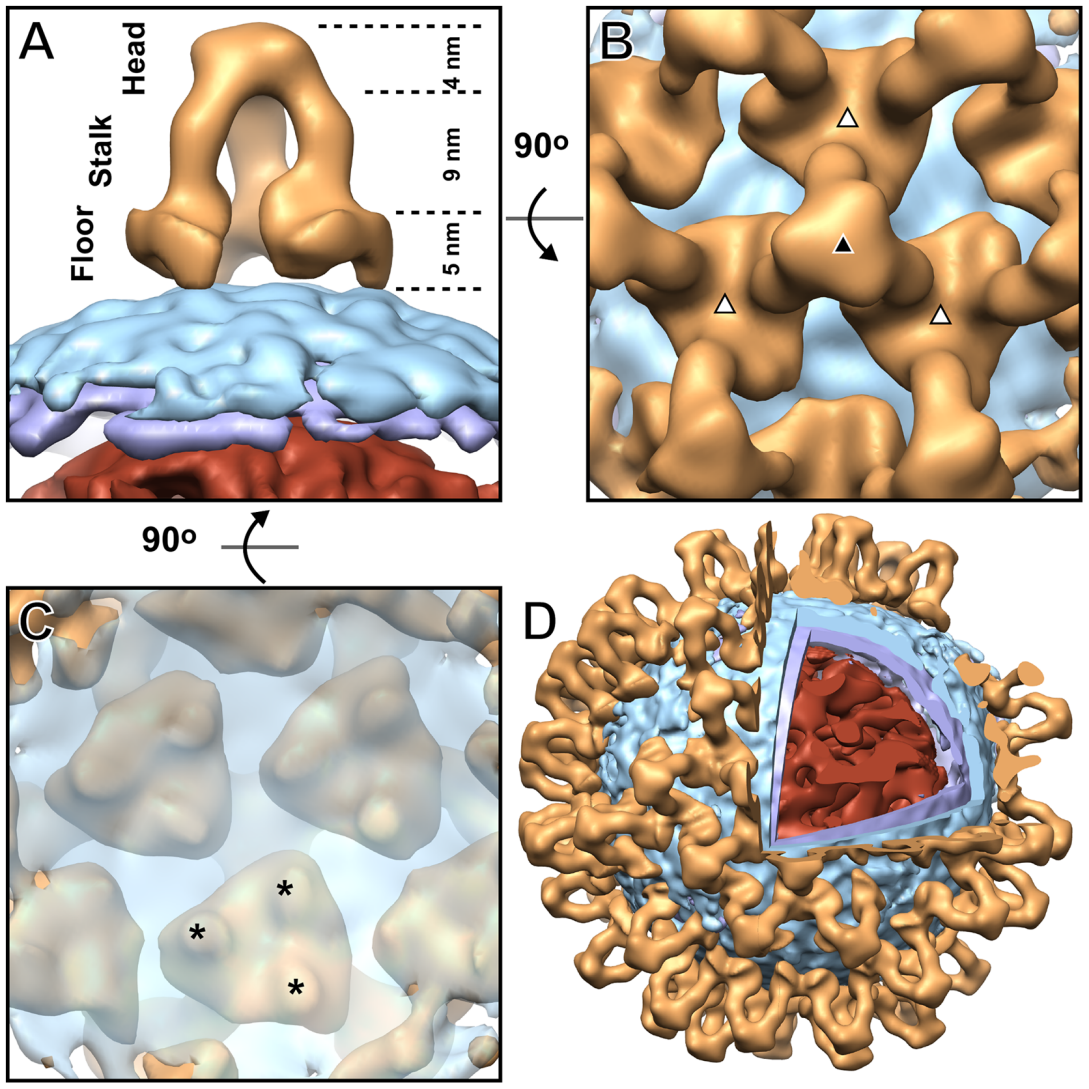
Tripodal architecture of BUNV glycoprotein spike. (A–C) The glycoprotein spike is shown from the side *A*, top *B*, and below the membrane *C*. The glycoprotein is shown in orange, the membrane outer leaflet in cyan, the inner leaflet in purple, and the RNP in red. Structural regions and their corresponding dimensions are indicated in *A*. The three-fold symmetry axis is indicated for an intra- (black triangle) and inter-trimeric (white triangle) interface in *B*. Stars indicate membrane–glycoprotein contacts in *C*. Each contact most likely corresponds to a transmembrane region shared between Gn and Gc. The glycoprotein was rendered at 1.5 sigma and the membrane and RNP at 1 sigma above the mean density. (D) A cut-open model of a BUNV virion generated by mapping the averaged structure of the glycoprotein spike onto the corresponding tomographic reconstruction. Coloring as in *A*. The glycoprotein was rendered at 1.5 sigma, the membrane leaflets at 1 sigma, and the RNP at 0.5 sigma above the mean density.

The glycoprotein spike, which we propose to correspond to a trimeric assembly of Gn–Gc heterodimers, forms a novel tripodal architecture (Figure 3). As calculated from the volume of the reconstruction, the estimated molecular mass of the glycoprotein spike is approximately 125–230 kDa, consistent with a molecular mass of a putative Gn–Gc heterodimer (∼140 kDa). Each protomer of the spike is elongated, extending 18 nm from the virion surface, and spanning through a ‘stalk’ region between two major protein–protein contacts: one located membrane proximally, which we refer to as the ‘floor’ region, and one located membrane distally, which we refer to as the ‘head’ region. Each of these protein–protein contacts is three-fold symmetric (Figure 3B) and was observed without imposing three-fold symmetry upon the reconstruction (Figure S3).

The floor region of the BUNV glycoprotein spike extends ∼5 nm above the viral membrane and forms a flat, triangle-shaped base (Figure 3B and C). Given the relatively small mass of the predicted ectodomain of the Gn glycoprotein (23 kDa), added to the presence of a C-terminal transmembrane region which anchors it to the virus envelope, we suggest that the Gn glycoprotein is localized to this area. From this deduction, we also propose that the considerably larger ectodomain of the Gc glycoprotein (103 kDa), binds to Gn in the floor region and solely corresponds to the density of the stalk and head regions.

### Tripodal Spikes Form Locally Ordered Patches Which Encapsulate the Virion

**Figure 4:**
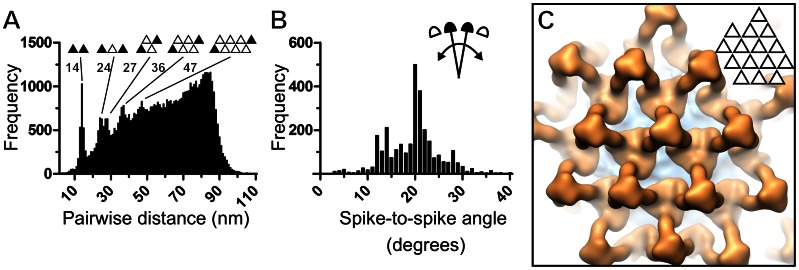
Locally ordered BUNV glycoprotein spike lattices. (A) Histogram of pairwise distances between glycoprotein spikes in the virions. The distance between corresponding trimers (black triangles) is indicated for the first five major peaks. (B) Histogram of angles between neighboring spikes with respect to the curvature of the membrane. (C) Close-up of virus surface showing a locally ordered lattice of 15 glycoprotein spike trimers generated by mapping the averaged structure of the glycoprotein spike onto the corresponding tomographic reconstruction.

In our structure of the glycoprotein spike, trimeric neighbouring spikes were also resolved, suggesting the presence of locally ordered lattices (Figure 2B, 3B, and S3A). To assess the extent of these repeating units, spike locations were mapped in 29 virions (Figure 3D and 4). Areas devoid of spikes were present between the locally ordered patches but usually these areas were too small to accommodate the tripodal spike structure. As an important caveat of this analysis, we cannot exclude the presence of a low population of alternative structures (e.g. monomeric spikes) or that some spikes were undetected. This analysis revealed that BUNV displays 91±4 spikes, putatively corresponding to 274±11 Gn–Gc heterodimers. Notably, this number is less than half of that estimated by early electrophoretic analysis of the related LACV [33].

When mapped onto tomographically reconstructed virions, locally ordered patches of spikes were observed where neighboring spikes were separated by 14 nm with up to four spikes in a row (Figure 4A and C). The volume between neighbouring spikes is of sufficient size to render the Gn and floor region fully accessible to functional cellular receptor(s) (Figure S5). The angle between neighboring spikes with respect to the curvature of the membrane was approximately 20 degrees (Figure 4B). Given the size of BUNV particles, it is plausible that this angle is sufficient to generate the curvature required for virus budding. The presence of locally ordered patches on the virion surface is reminiscent to Tula hantavirus (TULV) [29], where tetrameric spikes form curved, locally ordered patches.

Is it possible that the locally ordered patches of spikes assemble to form an icosahedrally symmetric configuration? The presence of two-fold symmetry axes is a prerequisite for an icosahedrally symmetric capsid built from trimers. However, we do not detect any two-fold symmetry. Firstly, in our averaged structure, we can detect only three-fold symmetry within and between trimers (Figure 3B; see also 2B, 4C, and S3A). Secondly, visual examination of all the 29 virions present in our data showed no signs of icosahedral symmetry. Together with the pleomorphic appearance and variation in size of BUNV virions in our electron micrographs and tomographic reconstructions (Figure 1), these observations strongly suggest that BUNV is not icosahedrally symmetric.

### Acidification Results in Altered Glycoprotein Structure

**Figure 5:**
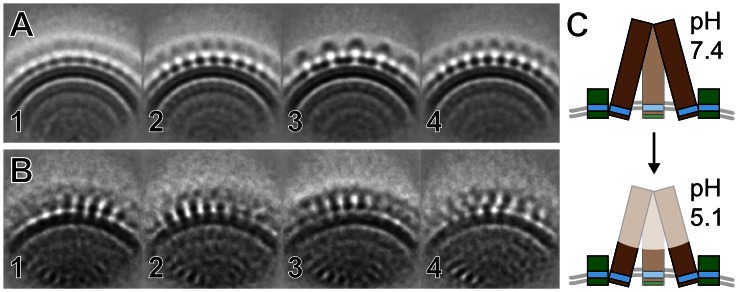
Low-pH induced structural rearrangements of glycoprotein spikes. (A and B) The first four class averages of glycoprotein spikes in neutral (pH 7.4) and acidic (pH 5.1) buffers are shown in panels *A* and *B*, respectively (all class averages are displayed in Figure S6). (C) Schematic illustration of pH-induced loss of order in the stalk and head domains of BUNV spike. Gn is colored green, Gc brown, transmembrane regions blue, and membrane gray.

Similar to phleboviruses [34], orthobunyavirus fusion glycoproteins have been predicted to exhibit a class-II fusion structure and are known to utilize clathrin-mediated endocytotic pathways for host cell entry [16], [17], [19], [20]. X-ray crystallographic investigations have shown that class-II fusion glycoproteins undergo significant conformational rearrangements upon acidification [35], [36]. We sought to determine whether BUNV fusion is consistent with this model by detecting conformational changes upon introduction to a fusion permissive acidic pH (Figure 5). We collected images of 286 virions, vitrified in acidic buffer (pH 5.1), and compared them with images of 428 virions at neutral pH (pH 7.4). Smaller extracted images of the spikes were subjected to 2-D alignment followed by classification by multivariate statistical analysis. Consistent with our 3-D structure derived from sub-tomogram averaging, 2-D class averages resulting from analysis of pH-neutral BUNV revealed repeating arrangements of spikes. As observed in our 3-D structure, protein–protein contacts were easily discernible at both membrane-proximal and -distal regions (Figure 5A and S6A). An equivalent analysis performed with glycoprotein spikes vitrified at pH 5.1 revealed a clear alteration in structure where the interconnecting protein lattice was disrupted and no longer visible in the acidic environment (Figure 5B, C, and S6B). Due to this disruption, we were unable to interpret the oligomeric properties of the BUNV glycoproteins in their post-fusion state. Despite these limitations, these data are suggestive of a significant conformational change in the structure of the putative Gc component of the glycoprotein spike. This is consistent with the insertion of hydrophobic fusion peptides into host cell membranes during endosomal trafficking and entry, a mode of action observed in viruses with class-II fusion glycoprotein folds [35], [36].

### Comparison of Glycoprotein Arrangements within the *Bunyaviridae*

**Figure 6:**
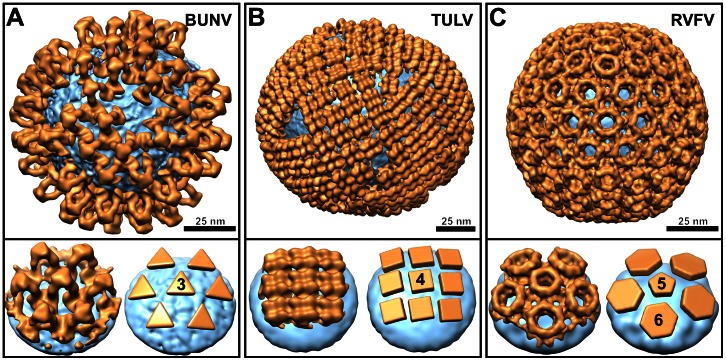
Structural diversity of bunyavirus surface glycoprotein architectures. (A–C) Structures of Gn–Gc glycoprotein spikes (orange) are shown mapped onto the membrane surface (cyan) of: (A) an orthobunyavirus (Bunyamwera virus; BUNV), (B) a hantavirus (Tula virus; TULV; EMD-1704^11^), and (C) a phlebovirus (Rift Valley fever virus; RVFV; EMD-1550^7^). At the bottom of each panel, we show a close-up view of a glycoprotein spike cluster (left) and a schematic representation of the spike arrangement (right). Symmetries of individual spikes are annotated.

The size and sequence of bunyaviral Gn and Gc glycoproteins, required for host cell entry, vary substantially between the five genera of this family [1]. It is now evident that this diversity is also reflected in structure (Figure 6). The glycoproteins of hantaviruses such as Hantaan virus [30] and TULV [29], for example, form tetrameric square-like arrangements which are unevenly distributed across the virion surface (Figure 6B). Phleboviruses such as RVFV [24], [25], [37] and UUKV [26], on the other hand, construct pentamers and hexamers capable of assembling into icosahedrally symmetric particles (Figure 6C). Our orthobunyavirus structure compounds this structural diversity, forming a bridging tripodal assembly (Figure 6A), not observed previously.

### BUNV Gc Structure and Different Classes of Fusion Machinery

Entry into a host cell by a membrane-containing virus relies on the attachment to host cell receptor(s) and subsequent fusion of host and viral membranes. These functions are often performed by two or more discrete proteins, which are synthesized on the same polypeptide and fold to form higher order assemblies on the virion surface. The folding architectures of viral attachment glycoproteins are varied, allowing binding to a diverse array of cell surface receptors [38], [39]. In contrast, fusion glycoproteins are more constrained in function, solely targeting the host cell envelope and exhibiting structural organization and assembly which is limited to three classes [35], [40].

Is the orthobunyaviral fusion glycoprotein compatible with known classes of fusion machinery? The elongated shape of the Gc glycoprotein is consistent with previous investigations which proposed the Gc of the closely related orthobunyavirus, LACV (63% similarity with BUNV over the M segment), to be a class-II fusion glycoprotein [19]. Indeed, the extended stalk region of BUNV Gc is consistent with the ‘rod-like’ pre-fusion architecture of class-II fusion glycoproteins such as the E glycoprotein from Dengue virus (DENV) [41] and tick-borne encephalitis virus (TBEV) [42]. The predominant β-stranded secondary structure predicted throughout the BUNV Gc ectodomain is also a conserved feature of class-II fusion architecture [35].

We also note differences between the dimeric, pre-fusion class-II assembly and our structural model. The pre-fusion state of BUNV Gc is trimeric, dissimilar to the anti-parallel arrangement of dimers observed in DENV E and TBEV E proteins. Indeed, an anti-parallel arrangement of dimers in the tripodal BUNV structure is not physiologically possible as this would place some of the Gc transmembrane regions distal from the viral membrane.

The trimeric arrangement of BUNV Gc also contrasts class I and III fusion glycoproteins. Structural studies of these glycoproteins in their pre-fusion state have revealed extensive protein–protein interfaces between protomers [43], [44]. These properties juxtapose the tripod-like arrangement of our BUNV spike, where protein–protein contacts are more minimal and exist solely at the floor and head regions (Figure 3B). This supports a unique mode of fusion glycoprotein oligomerization, dissimilar to that observed in the currently known classes of fusion glycoproteins.

### Conclusions

In this study, we show that orthobunyaviruses constitute a structurally unique group. Through analysis of BUNV and comparison with previous analyses of LACV, we reveal that orthobunyaviruses are pleomorphic and contrast the icosahedral organization observed in phleboviruses. The glycoprotein spike, which we propose to be composed of three Gn–Gc heterodimers, forms a tripodal architecture which further assembles to form locally ordered patches encapsulating the virion. These patches are conceptually analogous to the tetrameric lattices observed in hantaviruses. The tripodal BUNV glycoprotein architecture is also pH-dependent. Upon acidification, we observe a major structural rearrangement in the head region of the trimeric spike, corresponding to the Gc fusion glycoprotein. Taken together, these results provide the first detailed 3-D structural analysis of an orthobunyavirus and a model for this large group of medically important viruses.

## Materials and Methods

### Virus Preparation

BHK-21 cells were maintained in Glasgow minimal essential medium (Invitrogen, Paisley, UK) supplemented with 8% tryptose phosphate broth and 10% newborn calf serum at 37°C in a humidified atmosphere containing 5% CO_2_. Cells were infected with BUNV at a multiplicity of infection of 0.01–0.1 and the medium containing excreted viral particles was collected forty hours after infection. BUNV particles were purified by pelleting through a sucrose cushion, resuspended in either neutral pH (7.4) or acidic, fusion permissive pH (5.1) [16] buffer and vitrified by plunge-freezing on C-flat EM grids (Protochips, Raleigh, NC) into a mixture of liquid ethane (37%) and propane (63%) [45]. Colloidal 10-nm gold particles coupled to bovine serum albumin were added for electron cryo-tomography. Sample purity was confirmed by SDS-PAGE analysis (Figure S1).

### Electron Cryo-microscopy and Image Processing

Electron cryo-microscopy was performed at liquid nitrogen temperature using a 300-keV transmission electron microscope (Polara; FEI, Eindhoven, Netherlands). Images of BUNV particles at pH 7.4 and pH 5.1 were taken at −4 to −5.5 µm defocus using a CCD camera (Ultrascan 4000SP; Gatan, Pleasanton, CA) at a calibrated magnification of ×75,000, corresponding to a pixel size of 0.20 nm with a dose of approximately 20 e^−^/Å^2^. 103 images were taken for pH 7.4, corresponding to 428 virions, from which 19,733 overlapping smaller images were extracted at 5 pixel intervals in 120×120 pixel boxes. 135 images were taken at pH 5.1, corresponding to 286 virions, from which 15,176 overlapping smaller images were extracted similarly. Pre-processing and contrast transfer function (CTF) correction were carried out in XMIPP [46]. Virion diameter was measured manually in the Bsoft software package [47], [48]. To calculate class averages, both datasets were treated identically. Images were rotationally pre-aligned in Bsoft [47], [48] and further aligned in IMAGIC [49]. To align the spike and membrane layers, vertical alignment of +/−24 pixels was performed and followed by rotational alignment of +/−10 degrees. The process was iterated three times using the average of all images as a reference. The aligned images were subjected to multivariate statistical analysis and hierarchical classification to 16 classes.

### Electron Cryo-tomography

Single-axis tilt series were collected over an angular range of −60° to 60° at 3° increments using the program SerialEM under low-dose conditions [50]. An energy filter (GIF 2002; Gatan, Pleasanton, CA) was operated in the zero-energy-loss mode with a slit width of 20 eV. Images were acquired at −4 to −4.5 µm defocus at a calibrated magnification of ×111,000, corresponding to a pixel size of 0.54 nm after binning by a factor of two. The total dose per each tilt series was approximately 100 e^−^/Å^2^. The IMOD package was used to calculate 3-D reconstructions [51]. For image alignment, 10-nm gold particles were used as fiducial markers. A low-pass filter was applied to each tomographic reconstruction to remove spatial frequencies higher than the first zero in the CTF of the microscope (*e.g.* −4.5 µm defocus corresponds to 1/3.0 nm). Twenty-nine spherical virions (360×360×360 voxels) were extracted from the tomographic reconstructions for further analysis.

### Generating an Initial Template Structure by Sub-tomogram Averaging

The membrane-bound Gn–Gc glycoprotein spike complex was resolved by iterative template-matching, alignment, and averaging as implemented by the programs *jviews*, *jsubtomo*, and *jave*, in the Jsubtomo software package (www.opic.ox.ac.uk/jsubtomo) [29], [52], [53]. An initial structure was calculated from approximately 750 manually picked spikes. Spike orientation was estimated in *jviews* exploiting the roughly spherical geometry of the virions. Alignment and averaging of spike location and orientation was performed with *jsubtomo* and iterated at each stage of refinement until convergence. The ‘missing wedge’ in the tomographic data, due to the incomplete tilt range in data collection, was taken into account in the correlation and the averaging. Firstly, the constrained cross-correlation (in the frequency range of 1/4.0 to 1/40 nm) between the template and each of the sub-volumes was calculated by applying a reciprocal space, wedge-shaped mask, reflecting the tilt geometry of −60° to 60°. Secondly, the program *jave* was used to calculate a missing-wedge weighted average (80×80×80 voxels) by normalizing under-represented areas in the Fourier space of the average. Spikes with the highest cross correlation (75%) were included in the average. Structural refinement consisted of three stages, as described below.

In the first stage of refinement, the direction of the view vector was refined (angles θ and φ). A spherical mask (35 nm in diameter), which included a central spike and contributions from the membrane and neighbouring spikes, was applied. Initial orientations were allowed to change by 32 degrees (in 8-degree increments) and locations by 10 pixels. Cylindrical symmetry was applied to the average following each iteration of refinement. In the second stage, the angle around the spike symmetry axis (α angle) was allowed to change up to 180 degrees, in 8-degree increments. An ellipsoidal mask slightly larger than the central spike was applied. The direction of the view vector (θ and φ) and location of each spike was fixed. No symmetry was assumed nor applied. In the third stage, three-fold symmetry was imposed (see “Symmetry Analysis of the Glycoprotein Spike”). The α angle was further refined by allowing changes of up to 60 degrees, in 8-degree increments. Three-fold symmetry was applied on the average following each iteration.

### Template Matching and Structure Refinement of Glycoprotein Spikes

The initial averaged structure, generated from the manually picked subset of spikes, was used to locate the spikes in all of the BUNV particles and to refine the structure to higher resolution. We adopted the ‘gold standard’ scheme proposed for single-particle analysis [54] for sub-tomogram refinement [55]: The refinement was carried out for two fully independent half sets of data to prevent over-refinement and to allow proper assessment of resolution by FSC. The search was restricted to the surface of each virion by placing evenly distributed and oriented pseudo-particles, or ‘seeds’, at the correct radius using *jviews*. Seeds (106 for each virion) were added every 20 degrees, corresponding to seed-to-seed distances of 28 pixels. To minimize bias caused by the initial tempate structure, the structure was first filtered to 8-nm resolution in Bsoft and then iteratively refined at each seed location using *jsubtomo*. Shifts of 14 and 30 pixels were allowed parallel and orthogonal to the plane of the membrane, respectively. The α angle was allowed to change by 60 degrees and θ and φ angles by 32 degrees, in 8-degree increments. Multiple hits corresponding to the same spike were discarded. This allowed the detection of the best correlating spike close to each seed location. To allow the average of all detected spikes to iteratively refine to a higher resolution, an adaptive band-pass filter was applied, where the high resolution cut off was 1.2 times the resolution of the averaged structure; the low resolution cut off was 10 times the high resolution cut off. The resolution of each averaged structure was determined by FSC, calculated between two maps generated from two independent half-sets of the data using a threshold of 0.143 [56]. The alignment converged in four iterations. Approximately 2,000 of the total of ∼2, 800 spikes (∼75%) were used to calculate the final averaged map. Between 1/135 Å and 1/30 Å spatial frequency, the least frequent Fourier term was observed 870 times and the most frequent Fourier term was observed 1,632 times.

### Symmetry Analysis of the Glycoprotein Spike

To detect symmetry in the unsymmetrized average (Figure S3A), we calculated a rotational self-correlation function in Dynamo package [57] between the averaged structure and the same structure rotated in one-degree increments around the long axis. The resulting plot revealed three clear maxima (−120°, 0° and 120°), consistent with three-fold symmetry (Figure S3B). The presence of three-fold symmetry was further quantified by Fourier transformation of the rotational self-correlation function in Matlab. The rotational power of the zero-term was normalized to a value of 1.0 (Figure S3C).

### Estimation of Glycoprotein Molecular Mass

To estimate the molecular mass of the putative heterodimeric Gn–Gc protomer in our reconstruction, density corresponding to one spike was rendered in UCSF Chimera at thresholds ranging between 3.5 and 1.5 sigma above the mean density. Assuming an average protein density of 0.81 Da/Å^3^, the volume of the rendered surfaces corresponded to a molecular mass between 125 kDa and 230 kDa.

### Mapping the Glycoprotein Spikes

To generate models of the BUNV virions for visualization (Figure 3), a single spike from the final averaged map was placed into each of the detected positions on the virions using *jsubtomo*. Composite maps were generated using *jave*, taking into account possible overlaps of rotated and shifted sub-volumes. 91±4 spikes nearly fully covered the virions, confirming that enough seeds (106 per virion) were used to locate all of the spikes.

A tomographic reconstruction of the Tula hantavirus virion and an averaged structure of the spike (EMD-1704) [29] were used to visualize the hantavirus glycoprotein spikes. A single particle reconstruction of Rift Valley fever virus (EMD-1550) [24] was used to visualize the arrangement of the phlebovirus glycoprotein spikes. Spike detection and mapping was carried out similarly to BUNV.

### Structure Validation by Comparison to Structures Derived by Single Particle Averaging

The structure of the BUNV glycoprotein spike was validated by comparison to structures derived by single particle averaging of spike side views using RELION (Figure S4) [58]. In contrast to the 2-D classification of spike side views that was carried out for the comparison of pH 7.4 and pH 5.1 data, pre-alignment was not performed and the entire alignment and reconstruction was carried out in RELION. Equidistantly-spaced (∼6 pixels) coordinates were defined at a constant radius of 45 nm for 109 images of nearly spherical virions from 42 micrographs taken between defocus values of −4.1 and −4.5 µm. CTF estimation was carried out in CTFFIND3 [59]. A total of ∼13,000 side-views were extracted (270×270 pixels) from micrographs and down-sampled by a factor of three, resulting in a final pixel-size of 6 Å and image size of 90×90 pixels. The initial reference, derived by sub-tomogram averaging, was low-pass filtered to very low resolution (60 Å) to avoid template bias at higher resolutions [58]. In addition to the correct symmetry (C3), two incorrect symmetries (C7 and C11) were applied on the starting model [30] to assess the robustness of the single particle approach (Figure S4A). During iterative alignment and averaging, the correct symmetry (C3) was imposed. Estimates for the two Euler angles (tilt and psi), derived from the nearly spherical geometry of the sub-set of particles used here, were treated as priors with Gaussian sigma value of 7 degrees. For comparison to the 30-Å resolution structure derived by sub-tomogram averaging, the refinement here was restricted to 30 Å. This resolution was reached in all refinements. Refinements converged after 16–20 iterations.

### Accession Numbers

The structure of the membrane-bound Gn–Gc complex has been deposited in the Electron Microscopy Data Bank at the European Bioinformatics Institute (accession no. EMD-2352).

## Supporting Information

Figure S1 SDS-PAGE analysis of purified BUNV (lane 2). Protein bands correspond to the BUNV structural proteins: nucleoprotein (NP), Gn, Gc, and polymerase (L) protein are shown. The molecular mass marker (kDa) is shown (lane 1).
(TIF)

Figure S2 Histogram displaying the variation in diameter (nm) of BUNV and RVFV particles.
(TIF)

Figure S3 Three-fold symmetry of the Bunyamwera virus (BUNV) glycoprotein ‘spike’ cluster. (A) In the structure calculated without imposing three-fold symmetry, the center-most spike is trimeric. The neighboring spikes are resolved and also three-fold symmetric, indicating local order in the lattice. (B) A rotational self-correlation plot following reconstruction of the unsymmetrized structure with maxima at −120°, 0°, and 120°. (C) Rotational power spectrum of BUNV glycoprotein spike structure showing rotational power as a function of rotational symmetry.
(TIF)

Figure S4 Validation of the BUNV glycoprotein spike structure by comparison to structures derived by iterative 2D single particle averaging. (A) Results from three refinement runs are shown. The same low-pass filtered (60 Å) structure from sub-tomogram averaging was used as an initial template in each run, but different symmetry (C3, C7 or C11) was imposed in each case on the template. During the reconstruction, C3 symmetry was imposed. In addition to the final models, two intermediate averaged structures are shown for each run to illustrate the convergence of the iterative refinement to the correct structure, irrespective of the initial symmetry. (B) Comparison of the final averaged structure from single particle averaging (left; the same map as in top row of panel *A*) to the average from sub-tomogram averaging (right; the same map as in Figure 3B). Both maps have been rendered at 1.5 sigma above the mean density and are displayed at the same magnification. (C) An overlay of the structure derived from single particle averaging (surface) and the structure derived from sub-tomogram averaging (mesh) is shown from top (left) and side views (right). The isosurface threshold of the sub-tomogram structure (2.5 sigma above the mean) was adjusted to match the surface of the single particle structure (1.5 sigma above the mean) to illustrate their structural agreement.
(TIF)

Figure S5 To measure the accessibility of the floor regions to putative receptor binding domains, two spherical markers were placed in two cavities between the spikes, one (green) in a smaller cavity directly on top of the floor region and the other (magenta) in a larger cavity between the spikes and touching the sides of three neighboring floor regions. The diameters of the green and magenta markers were 44 Å and 114 Å, respectively. Top (A) and side (B) views are shown.
(TIF)

Figure S6 Class averages of BUNV glycoprotein spikes at pH 7.4 (A) and pH 5.1 (B). Each class constitutes between 800 to 1,100 members. Classes are sorted from best to worst, as defined by the overall class quality in Imagic.
(TIF)
