# Supplementary figures and images for: Orthobunyavirus Ultrastructure and the Curious Tripodal Glycoprotein Spike
Source: PLoS Pathog. 2013 May 16;9(5):e1003374. doi: 10.1371/journal.ppat.1003374 (PMC3656102; doi:10.1371/journal.ppat.1003374)

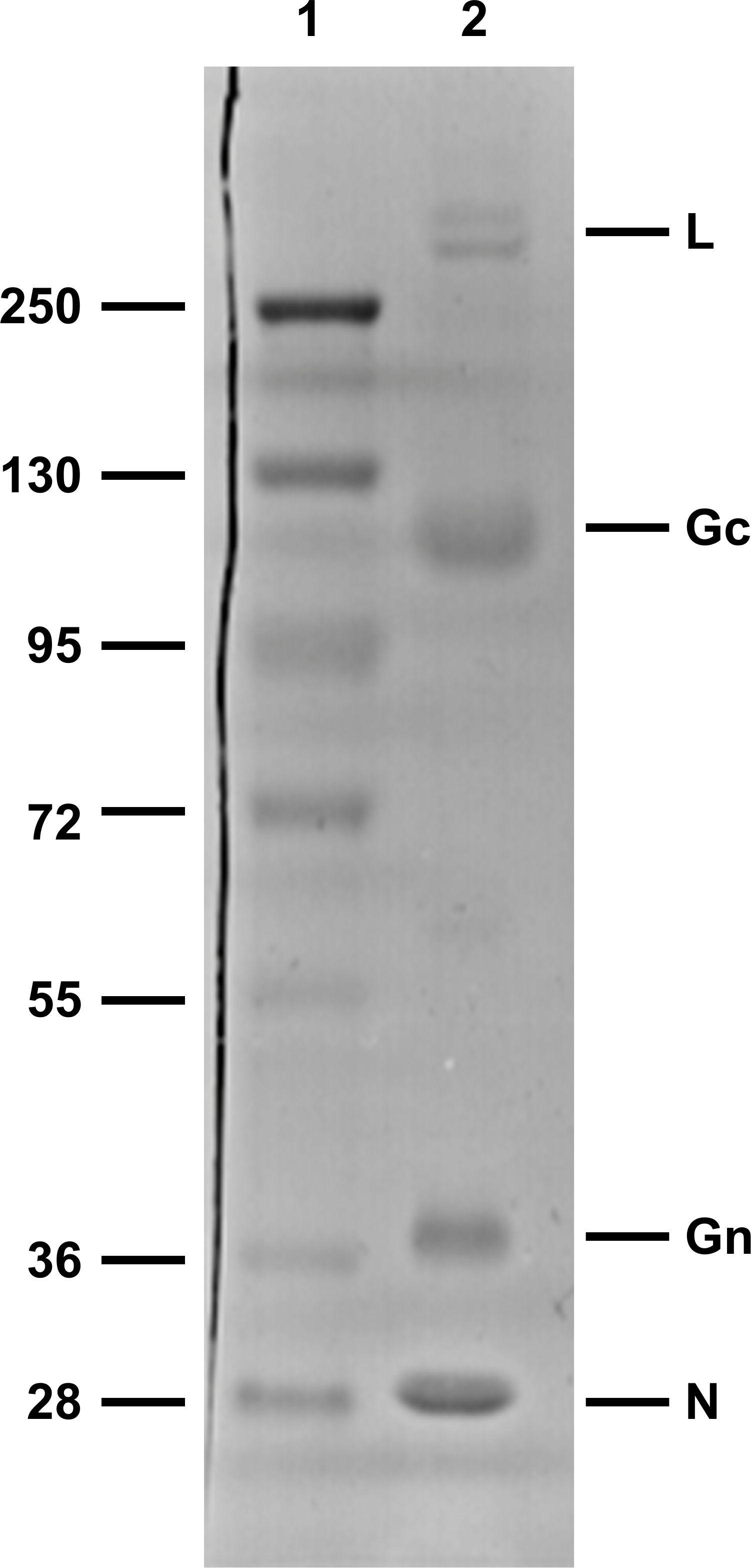

Supplement: Figure S1 — SDS-PAGE analysis of purified BUNV (lane 2). Protein bands correspond to the BUNV structural proteins: nucleoprotein (NP), Gn, Gc, and polymerase (L) protein are shown. The molecular mass marker (kDa) is shown (lane 1). (TIF) [file ppat.1003374.s001.tif]

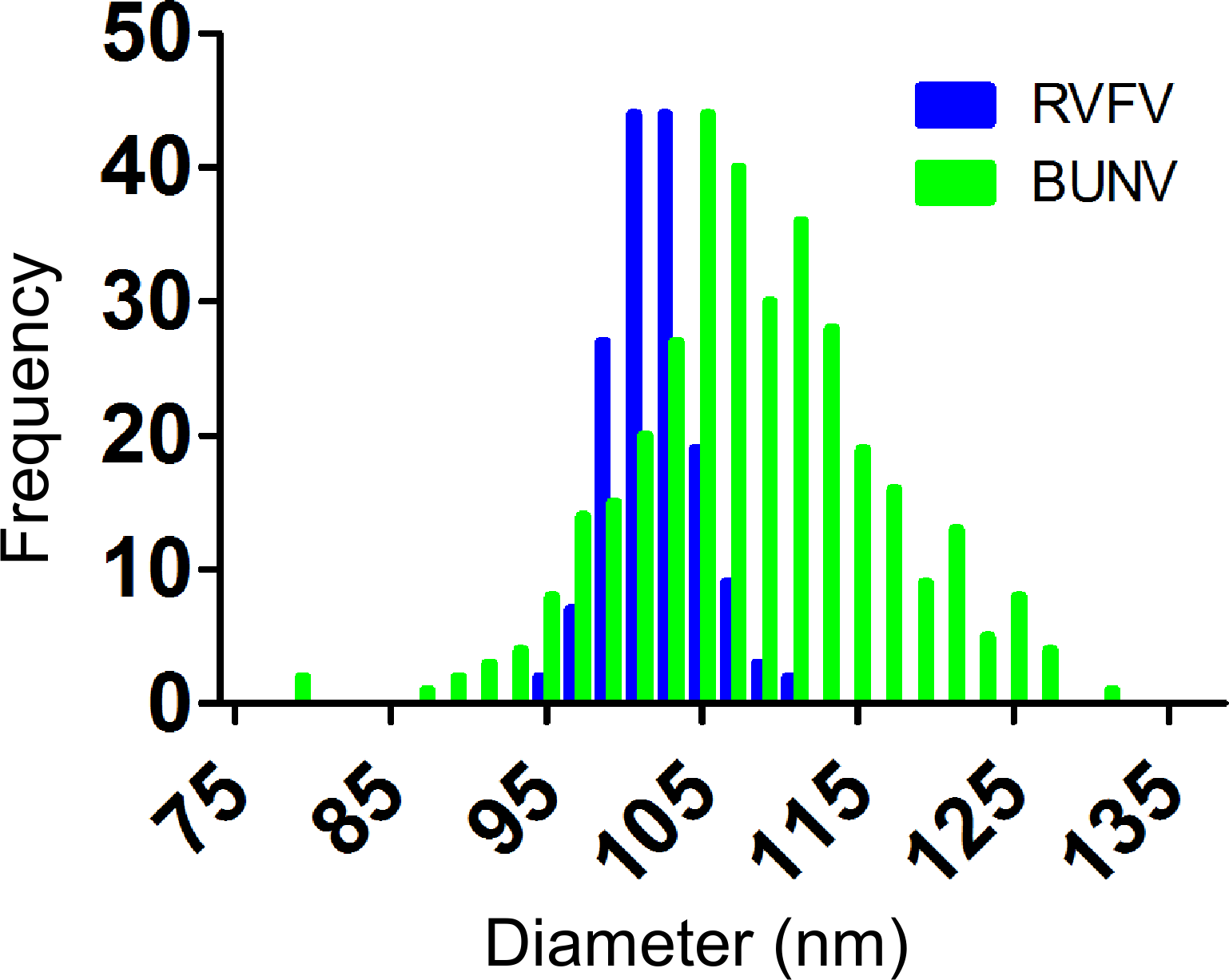

Supplement: Figure S2 — Histogram displaying the variation in diameter (nm) of BUNV and RVFV particles. (TIF) [file ppat.1003374.s002.tif]

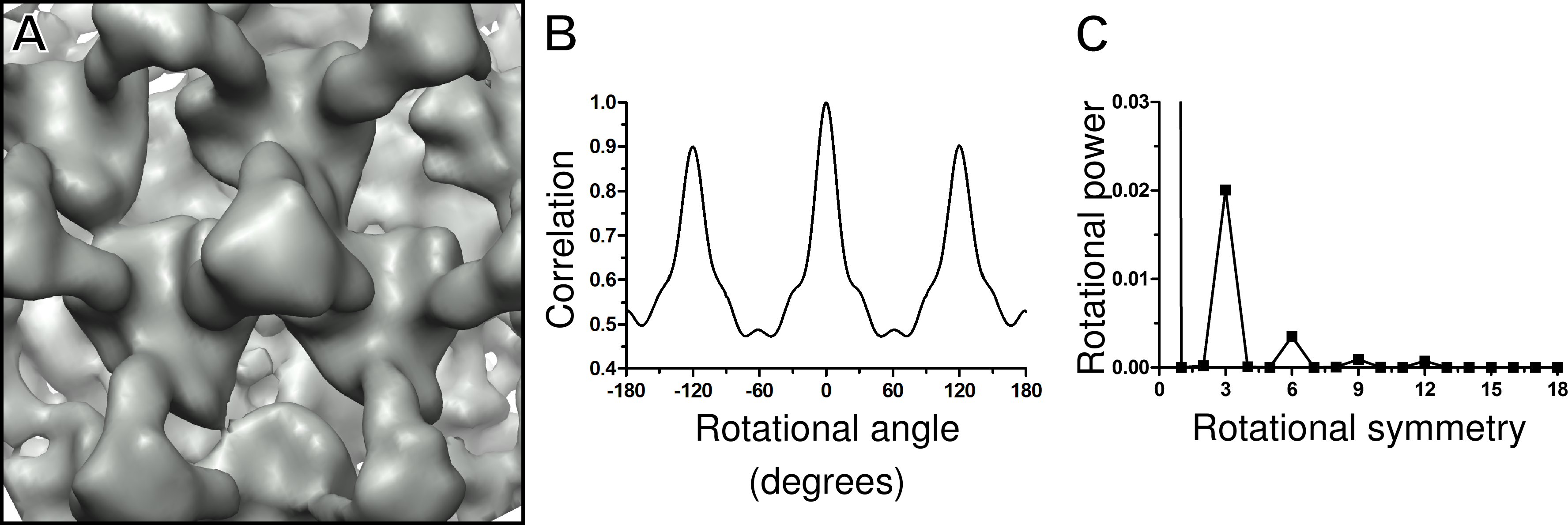

Supplement: Figure S3 — Three-fold symmetry of the Bunyamwera virus (BUNV) glycoprotein ‘spike’ cluster. (A) In the structure calculated without imposing three-fold symmetry, the center-most spike is trimeric. The neighboring spikes are resolved and also three-fold symmetric, indicating local order in the lattice. (B) A rotational self-correlation plot following reconstruction of the unsymmetrized structure with maxima at −120°, 0°, and 120°. (C) Rotational power spectrum of BUNV glycoprotein spike structure showing rotational power as a function of rotational symmetry. (TIF) [file ppat.1003374.s003.tif]

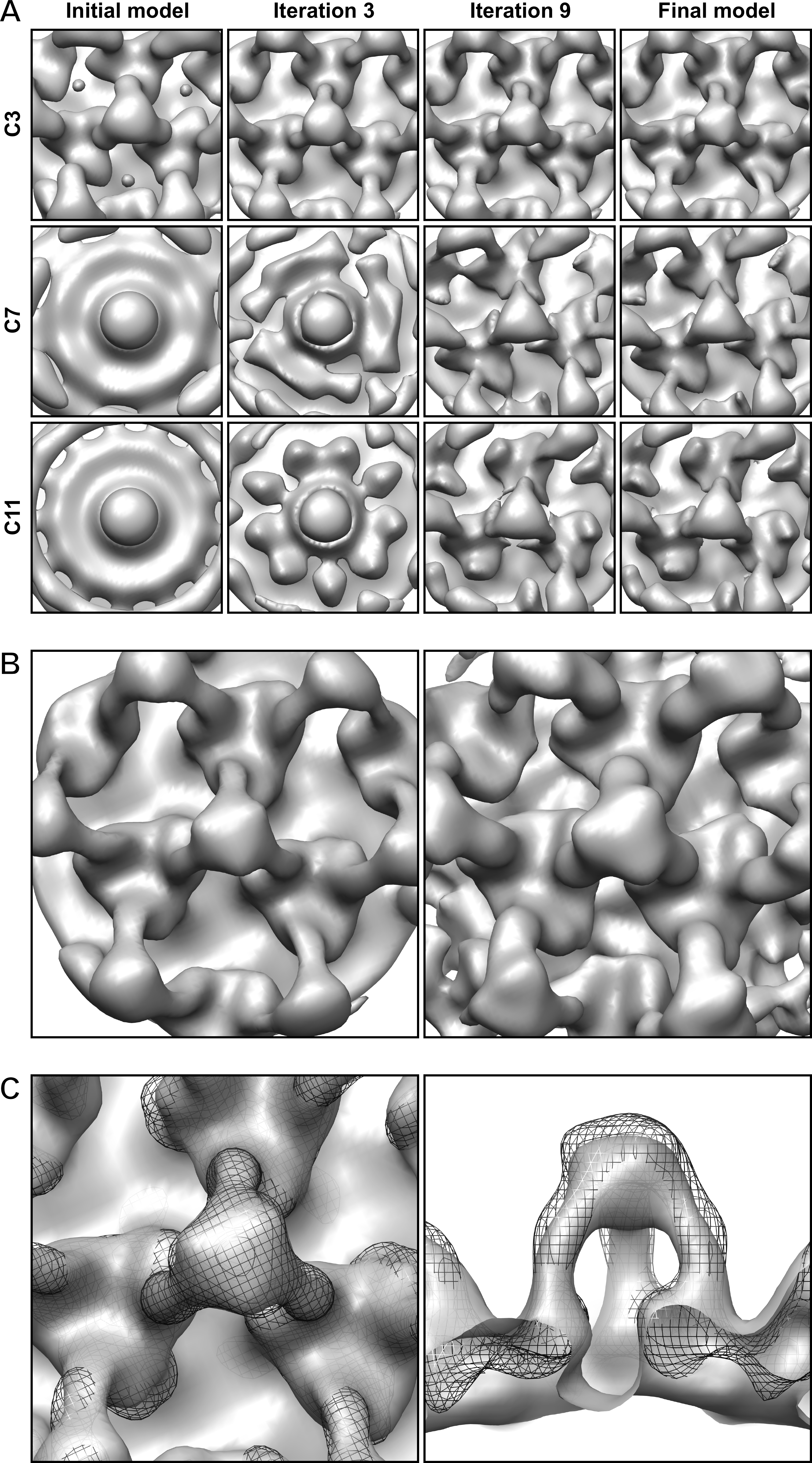

Supplement: Figure S4 — Validation of the BUNV glycoprotein spike structure by comparison to structures derived by iterative 2D single particle averaging. (A) Results from three refinement runs are shown. The same low-pass filtered (60 Å) structure from sub-tomogram averaging was used as an initial template in each run, but different symmetry (C3, C7 or C11) was imposed in each case on the template. During the reconstruction, C3 symmetry was imposed. In addition to the final models, two intermediate averaged structures are shown for each run to illustrate the convergence of the iterative refinement to the correct structure, irrespective of the initial symmetry. (B) Comparison of the final averaged structure from single particle averaging (left; the same map as in top row of panel A) to the average from sub-tomogram averaging (right; the same map as in Figure 3B). Both maps have been rendered at 1.5 sigma above the mean density and are displayed at the same magnification. (C) An overlay of the structure derived from single particle averaging (surface) and the structure derived from sub-tomogram averaging (mesh) is shown from top (left) and side views (right). The isosurface threshold of the sub-tomogram structure (2.5 sigma above the mean) was adjusted to match the surface of the single particle structure (1.5 sigma above the mean) to illustrate their structural agreement. (TIF) [file ppat.1003374.s004.tif]

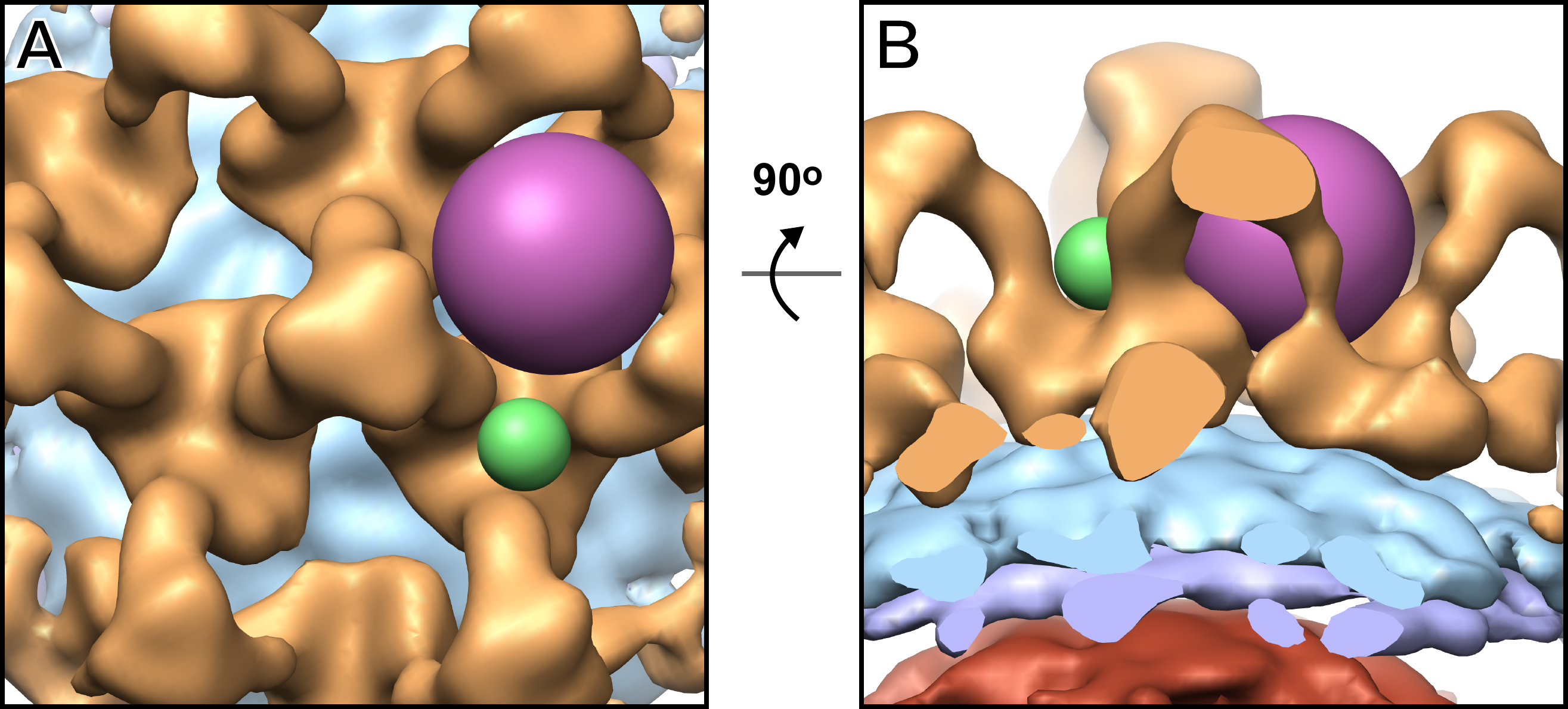

Supplement: Figure S5 — To measure the accessibility of the floor regions to putative receptor binding domains, two spherical markers were placed in two cavities between the spikes, one (green) in a smaller cavity directly on top of the floor region and the other (magenta) in a larger cavity between the spikes and touching the sides of three neighboring floor regions. The diameters of the green and magenta markers were 44 Å and 114 Å, respectively. Top (A) and side (B) views are shown. (TIF) [file ppat.1003374.s005.tif]

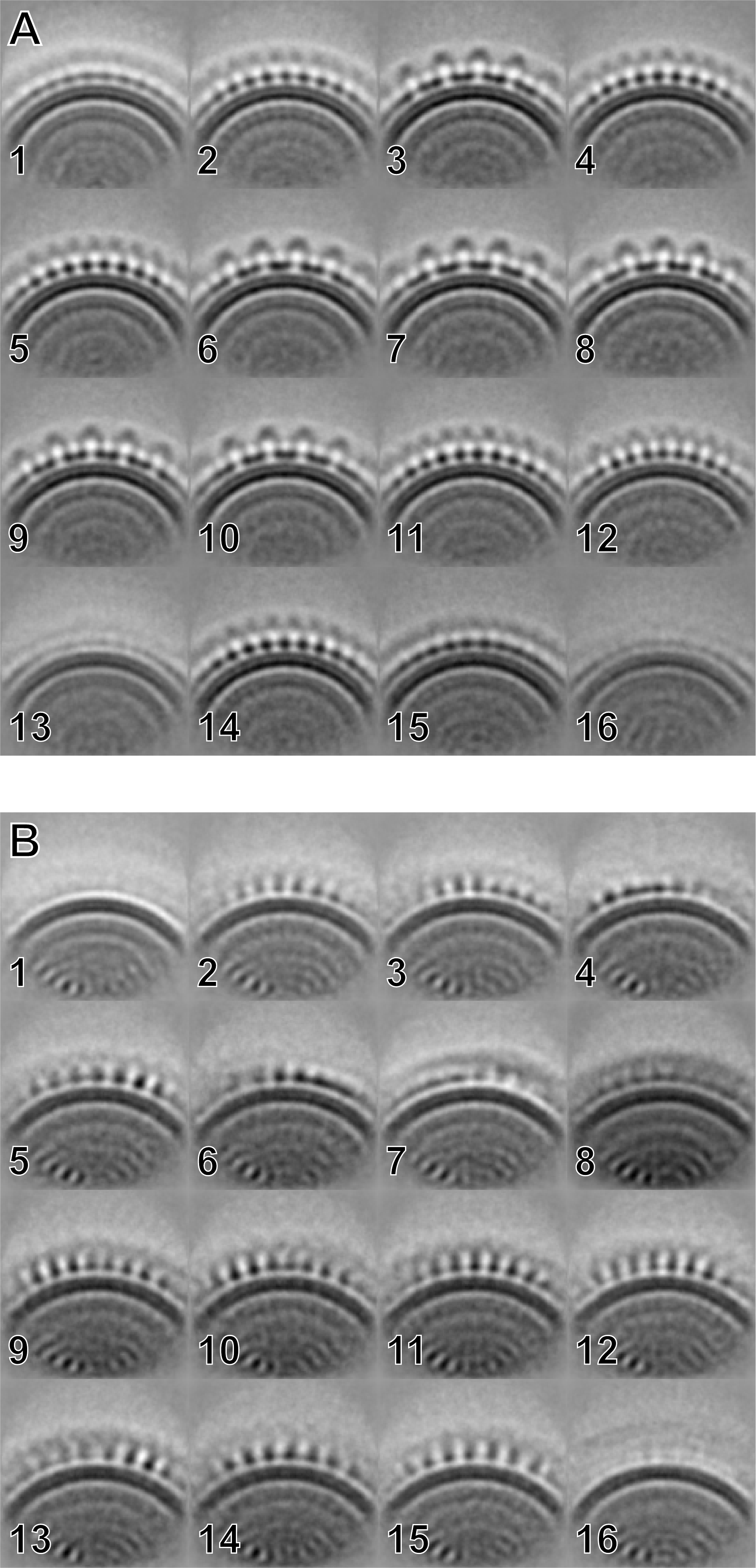

Supplement: Figure S6 — Class averages of BUNV glycoprotein spikes at pH 7.4 (A) and pH 5.1 (B). Each class constitutes between 800 to 1,100 members. Classes are sorted from best to worst, as defined by the overall class quality in Imagic. (TIF) [file ppat.1003374.s006.tif]
